# In silico analysis of local RNA secondary structure in influenza virus A, B and C finds evidence of widespread ordered stability but little evidence of significant covariation

**DOI:** 10.1038/s41598-021-03767-x

**Published:** 2022-01-10

**Authors:** Jake M. Peterson, Collin A. O’Leary, Walter N. Moss

**Affiliations:** Roy J. Carver Department of Biophysics, Biochemistry and Molecular Biology, Iowa State University, Ames, IA 50011 USA

**Keywords:** Computational biology and bioinformatics, Genetics, Microbiology

## Abstract

Influenza virus is a persistent threat to human health; indeed, the deadliest modern pandemic was in 1918 when an H1N1 virus killed an estimated 50 million people globally. The intent of this work is to better understand influenza from an RNA-centric perspective to provide local, structural motifs with likely significance to the influenza infectious cycle for therapeutic targeting. To accomplish this, we analyzed over four hundred thousand RNA sequences spanning three major clades: influenza A, B and C. We scanned influenza segments for local secondary structure, identified/modeled motifs of likely functionality, and coupled the results to an analysis of evolutionary conservation. We discovered 185 significant regions of predicted ordered stability, yet evidence of sequence covariation was limited to 7 motifs, where 3—found in influenza C—had higher than expected amounts of sequence covariation.

## Introduction

Influenza belongs to the segmented, single-stranded, negative-sense RNA *Orthomyxoviridae* family, occupying four genera (*Alphainfluenzavirus*, *Betainfluenzavirus*, *Gammainfluenzavirus*, and *Deltainfluenzavirus*). Of these, Alpha, Beta and Gamma are able to infect humans. Respectively, these genera consist of one species each: influenza A, B and C. Within each genome, influenza A virus (IAV) and influenza B virus (IBV) consist of eight viral RNA (vRNA) segments, and influenza C virus (ICV) consists of seven. IAV is considered the most threatening species to human health, possessing multiple antigenically distinct sub-types that can infect human and non-human hosts—allowing for the antigenic shifts that lead to global pandemics^1,2^. The deadliest example of this for influenza was in 1918, when an H1N1 variant killed an estimated 50 million people globally^3^. Due to this consistent threat, IAV has received a majority of our attention and resources; however, concerns over evolving lineages of IBV continue to mount^4^. This development has led to the current quadrivalent vaccines, which protect against two IAV strains and both main IBV lineages (Victoria and Yamagata)^5^. ICV is associated with mild respiratory symptoms in a majority of cases, however it has been found to cause serious illness in children^6,7^. All three clades are therefore worthy of additional study.

It is imperative to continue to take the lessons learned during prior pandemics and apply them toward future potential threats. Work from the first SARS outbreak led to a World Health Organization response model that was tested against the H1N1 outbreak^8^. Despite the considerable action taken prior to the outbreak, vaccine supply and distribution met a number of roadblocks that decreased efforts to slow its spread^8^. These impairments were improved upon and ultimately tested by the severe acute respiratory syndrome coronavirus 2 (SARS-CoV-2) pandemic. Development and approval time of SARS-CoV-2 vaccines were on an unprecedented time scale, becoming the new model for future pandemics^9^.

This new prevention model can be reapplied to influenza, however the biggest issue in influenza vaccine development is target availability. Current influenza vaccines involve an antibody response to the head domain of the hemagglutinin protein, one of two viral surface proteins^2,9^. The targeting of surface proteins is a common therapeutic approach, but influenza’s hemagglutinin is a particularly variable target, resulting in vaccines with short shelf lives and limited efficacy^9^. Advances in vaccine production (e.g., mRNA vaccines^9^) hold great promise in mitigating flu-related illnesses and death, yet there still remains a critical time gap between viral discovery and vaccine distribution. In that gap, it is necessary to have effective treatments for dealing with active infections. Only four drugs are FDA-approved for the treatment of influenza, with many influenza strains already evolving some level of drug-resistance^10^. Additional therapeutic modalities for treating influenza infections are therefore sorely needed.

One alternative approach would be the targeting of conserved RNA secondary structures critical to the viral life cycle. For example, recent work on enterovirus has found an RNA stem loop that undergoes a conformational change when an inhibitor is used, repressing translation^11^. This example is novel in that the 5ʹ UTR of the mRNA forms an internal ribosome entry site (IRES) to promote cap-independent translation, and that a small molecule library was used to effectively target RNA structure in the IRES^11^. Similar work was conducted against the SARS-CoV-2 frameshift stimulatory element, using small molecules to disrupt the secondary structure and inhibit a critical ribosomal frameshift^12^. While knowledge of what constitutes a “good” viral RNA target remains nascent, and there exist few examples within literature, it is imperative to develop a list of novel therapeutic targets using the tools currently available. With this in mind, it is useful to revisit and thoroughly define the influenza structurome to gain new insights on potential therapeutic targets.

Almost a decade ago, all three major clades of influenza were analyzed for conserved RNA secondary structural motifs in silico^13,14^. Subsequent experimental work focused on validation of local structural motifs^15–22^, testing their potential function^23^ and building global secondary structure models of their genomic vRNA^24^ and individual positive-sense RNAs^25^. More recent work using chemical crosslinking coupled to RNA-seq has focused on defining long-range intra- and inter-segmental RNA-RNA interactions that could be significant to genome packaging^26^. Despite this extensive in silico and experimental work on influenza structure/function, space remains for additional analyses—particularly those significant to drug discovery. This provided the motivation for our current study, where we apply the ScanFold pipeline to influenza virus. ScanFold is a program that divides the analysis of RNA secondary structure into two steps: firstly, long sequences are decomposed into multiple overlapping analysis windows, where each fragment is folded in silico and various thermodynamic properties are calculated; secondly, models of structure and predicted ordered biases in structure are combined to generate consensus base pairs that are weighted by their contribution to unusual ordered-stability. In this way, ScanFold provides local scans of the folding landscape across an RNA and discrete local motifs with high propensity for ordered (likely evolved) structural motifs^27^.

In contrast to previous analyses of influenza, which focused on individual windows and limited homology^14^, our current approach focuses on a single sequence and is able to define motifs (and their extent) in a robust and reproducible manner. For example, ScanFold has been successfully applied to analyze the genomes of Zika and HIV^27^, human herpes viruses^28^ and most recently to SARS-CoV-2—where models were used to rationally design a small molecule inhibitor of viral frameshifting^29^. An additional motivation for this current study is the analysis of conservation of motifs of interest. Most previous studies of influenza virus RNA secondary structure applied simple conservation metrics that were unable to define statistically significant covariation. In this current work we sought to assess structure-related sequence covariation using rigorous methods. Thus, by revisiting influenza with contemporary approaches, we hope to provide additional basic insights to guide investigations into influenza biology and to expand the list of potentially druggable RNA motifs in these viruses.

## Results

### Maps of local RNA structural propensity across influenza A, B and C

To generate maps of local secondary structural propensities, each segment and strand of IAV, IBV, and ICV was submitted to ScanFold-Scan for analysis (46 RNAs accounting for 81,892 nucleotides of sequence data scanned). IAV sequences (A/Puerto Rico/8/1934) were selected due to their prevalence in experimental studies, while IBV and ICV reference sequences (B/Lee/1940 and C/Ann Arbor/1/50, respectively) were selected to provide structural data applicable to the broadest range of viral targets. A scanning window of 120 nucleotides (nt) with a single nt step size resulted in over 75,000 almost fully overlapping (119 nt) analysis windows. For each window scanned, several key features were predicted: a minimum free energy (MFE) secondary structure and its associated change in Gibb’s folding free energy (ΔG°, a measure of thermodynamic stability); a thermodynamic z-score that compares the MFE ΔG° of the natively ordered sequence to the average ΔG° of 100 randomly shuffled versions of the sequence (z-score, the stability order-bias of the sequence); a partition function, from which is derived an ensemble centroid structure (best representative of the ensemble of probable conformations); and the ensemble diversity (ED, an indication of the volatility of the structural ensemble). Overviews of every RNA scanned are available in Supplemental File 1, and the raw data may be accessed on the RNAStructuromeDB (see “Data availability”).

**Figure 1:**
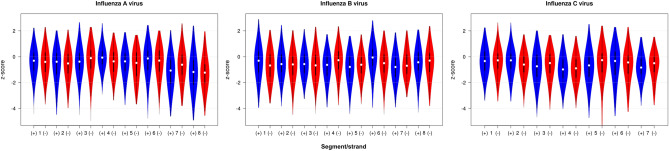
z-score violin plots of each influenza virus, with positive (blue) and negative (red) strands for comparison. While the range of z-scores observed is broad, there is a visual trend toward the negative (structured) across all clades, with IAV segments 7 and 8 being noticeably lower than other segments. Data from ScanFold, 120 nt window, 1 nt step, 100 randomizations, 37 ˚C. Image adapted from BoxPlotR^51^.

A summary of average ScanFold-Scan results for each clade, segment and strand can be found in Table 1. One of the key features of this analysis is the mapping of local z-scores across each influenza segment and strand using an approach adapted from Clote et al.^30^. The z-score metric is an indication of unusual ordered-stability, where negative values indicate the number of standard deviations more stable the MFE ΔG° of the natively ordered sequence is versus a pool of randomly shuffled sequences, which can indicate that a sequence has been driven by evolution to fold into a stable secondary structure. Alternatively, higher z-scores indicate a higher, less stable predicted MFE ΔG° versus the shuffled pool, signifying an evolutionarily driven region that breaks up native pairing contacts. A broad picture of the range of z-scores can be seen in Fig. 1. IAV had an overall average z-score (z_avg_) of −0.51 ± 1.14 and −0.53 ± 1.14 for the positive and negative strand, respectively, IBV had −0.56 ± 1.11 and −0.60 ± 1.07, and ICV had −0.63 ± 1.12 and −0.63 ± 1.08. The negative trend observed in these data indicate some potential for influenza being inherently structured and are in-line with previous predictions performed on influenza, which found similar skews in predicted z-score^31^.

**Table 1:** Average ScanFold metrics and extracted motifs for each strand.

Type & segment	z_avg_ & std dev	% z_avg_/nt	% z_avg_/nt	% z_avg_/nt	# < −2 motifs
	(+ / −)	< − 0 (+ / −)	< − 1 (+ / −)	< − 2 (+ / −)	(+ / −)
IAV 1	− 0.40 ± 0.98/− 0.44 ± 1.06	62.51/64.36	26.78/29.30	5.90/7.83	2/5
IAV 2	− 0.63 ± 1.20/− 0.63 ± 1.10	64.99/67.82	33.21/32.72	13.86/13.41	8/7
IAV 3	− 0.41 ± 1.05/− 0.26 ± 1.13	65.52/54.40	25.54/23.27	6.43/8.80	5/5
IAV 4	− 0.07 ± 0.84/− 0.42 ± 1.00	53.10/63.17	12.24/29.17	0.90/6.81	6/3
IAV 5	− 0.48 ± 1.05/− 0.62 ± 1.19	64.45/64.18	27.66/34.51	9.96/17.15	7/4
IAV 6	− 0.33 ± 1.18/− 0.40 ± 1.13	54.33/58.11	25.19/28.36	8.89/11.13	2/5
IAV 7	− 1.19 ± 1.23/− 0.69 ± 1.23	83.04/71.26	51.76/36.01	26.87/15.42	5/5
IAV 8	− 1.21 ± 1.42/− 1.24 ± 1.22	79.64/84.18	55.25/57.72	28.40/25.68	6/6
IBV 1	− 0.42 ± 1.20/− 0.69 ± 1.15	61.49/69.41	30.46/40.28	10.45/14.01	8/6
IBV 2	− 0.53 ± 1.03/− 0.71 ± 1.10	70.28/74.20	32.68/36.42	8.07/12.08	2/6
IBV 3	− 0.54 ± 0.96/− 0.64 ± 1.07	69.50/71.32	32.66/39.52	6.43/8.54	1/5
IBV 4	− 0.77 ± 1.05/− 0.35 ± 1.04	75.55/60.35	37.15/24.11	12.71/8.11	2/2
IBV 5	− 0.85 ± 1.08/− 0.66 ± 0.91	74.56/76.66	42.45/32.23	16.03/7.96	4/2
IBV 6	− 0.16 ± 1.13/− 0.57 ± 1.06	52.43/69.05	17.80/31.85	7.86/9.25	2/4
IBV 7	− 0.83 ± 1.05/− 0.58 ± 1.02	77.99/71.83	41.32/38.53	12.50/7.56	3/1
IBV 8	− 0.43 ± 1.11/− 0.46 ± 1.16	63.97/64.07	27.33/29.89	8.90/11.57	1/2
ICV 1	− 0.33 ± 1.02/− 0.39 ± 0.97	62.39/64.44	27.09/23.17	4.99/6.95	2/2
ICV 2	− 0.39 ± 0.97/− 0.64 ± 0.94	64.44/73.51	23.17/32.59	6.95/8.86	5/2
ICV 3	− 0.77 ± 1.18/− 0.60 ± 1.07	70.88/67.44	40.26/31.54	17.34/12.31	12/4
ICV 4	− 0.95 ± 1.03/− 0.91 ± 1.00	80.66/80.86	49.28/46.16	14.79/14.74	0/5
ICV 5	− 0.77 ± 1.21/− 0.56 ± 1.32	75.12/62.38	38.57/29.03	15.11/9.42	7/3
ICV 6	− 0.55 ± 1.34/− 0.66 ± 1.20	59.75/70.88	30.16/30.82	15.93/14.89	4/3
ICV 7	− 0.78 ± 0.93/− 0.69 ± 1.01	79.53/74.88	40.07/29.17	9.19/13.36	1/3

“% z_avg_/nt” is the percentage of nucleotides that had z_avg_ scores below the given threshold. “# < −2 Motifs” is the number of extracted motifs for each strand below the −2 z− score threshold, totaling 185.

Notably, only IAV positive segment 7 (−1.19 ± 1.23), IAV positive segment 8 (−1.21 ± 1.42), and IAV negative segment 8 (−1.24 ± 1.22) had z_avg_ below −1, indicating that they are globally ordered. Only these segments/strands approached the z-score values we^29^ (and others^32^) recently predicted for the genome of SARS-CoV-2 (average z-score −1.49^29^)—raising interesting questions about the potential roles of globally ordered RNA structure in each RNA. In SARS-CoV-2, a likely role is in genome packaging and post-transcriptional gene regulation, whereas in influenza, which consists of minus (−) sense vRNAs that are packaged and plus ( +) sense RNAs, the likely role is post-transcriptional control and genome replication/packaging. Potential evolutionary pressure to form structures useful in post-transcriptional gene regulation and packaging are likely different for the (−) versus the ( +) RNAs, but as these RNAs comprise sense/antisense pairs, such pressures are likely to have “echoes” across each strand. Thus, the forces working on influenza RNA structure are likely more complex than those of SARS-CoV-2.

Even in segments/strands without global z-score biases, however, significantly low regions were observed (Supplemental File 1). This can be assessed from the percentage of nucleotides per segment with z-scores below a given threshold, the % z_avg_/nt (Table 1). This latter metric was calculated in the second ScanFold stage, ScanFold-Fold (further discussed in the next section), where overlapping window z-score values are partitioned per nucleotide—giving a per-nucleotide metric to assess propensity for ordered stability. Here, it becomes more apparent that influenza is predominantly biased toward ordered structure, as a majority of nucleotides showed a predominant shift toward negative z_avg_/nt. Further lowering the z_avg_/nt threshold to below −2, the percentages range from a high of 28.40% for IAV 8 ( +) and a low of 0.90% for IAV 4 ( +). Interestingly, IAV 4 ( +) still had 6 predicted motifs with at least one unusually stable (< −2 z_avg_) base pair (bp). Potential implications of this are the existence of structure within influenza sequences, with varying degrees of structure across each segment. These regions were of particular interest, and were further analyzed to address this implication.

### Identification of local motifs with propensity for ordered stability and potential functionality

In the second stage of our analysis, ScanFold-Fold was used to identify the base pairs that most contributed to low z-score windows identified by ScanFold-Scan. This was accomplished by generating z-score weighted consensus structures where recurring base pairs in overlapping low z-score windows are favorably weighted. This resulted in numerous low z-score base pairs across influenza virus RNAs (listed in Table 1 and Supplemental File 1). A major feature of ScanFold-Fold is that z-score weighted consensus base pairs can be partitioned into discrete and unique local structural motifs. An example of these motifs can be seen at the 3ʹ end of ICV 5 (−), where three motifs are predicted in close proximity (1642–1678, 1684–1744, 1747–1800) (Supplemental File 1). While ICV 5 (−) has a z_avg_ of −0.56 ± 1.32, the range from 1642 to 1800 nt has a z_avg_ of −4.21 ± 0.63. This is the lowest z_avg_ observed for any predicted motif. The total number of motifs with at least one unusually stable bp was 185 across the 46 sequences motifs (all 185 motifs, locations, and structures are available in Supplemental File 2). Notably, ICV 3 ( +) had 12 motifs, 7 of which have z_avg_ below −2 (sequence and z_avg_, respectively: 328–353 nt, −2.09 ± 0.53; 357–403 nt, −2.29 ± 0.18; 908–988 nt, −2.34 ± 0.40; 1984–2006 nt, −2.10 ± 0.08; 2009–2023 nt, −2.40 ± 0.03; 2026–2066 nt, −2.51 ± 0.08; 2070–2129 nt, −2.33 ± 0.04).

**Figure 2:**
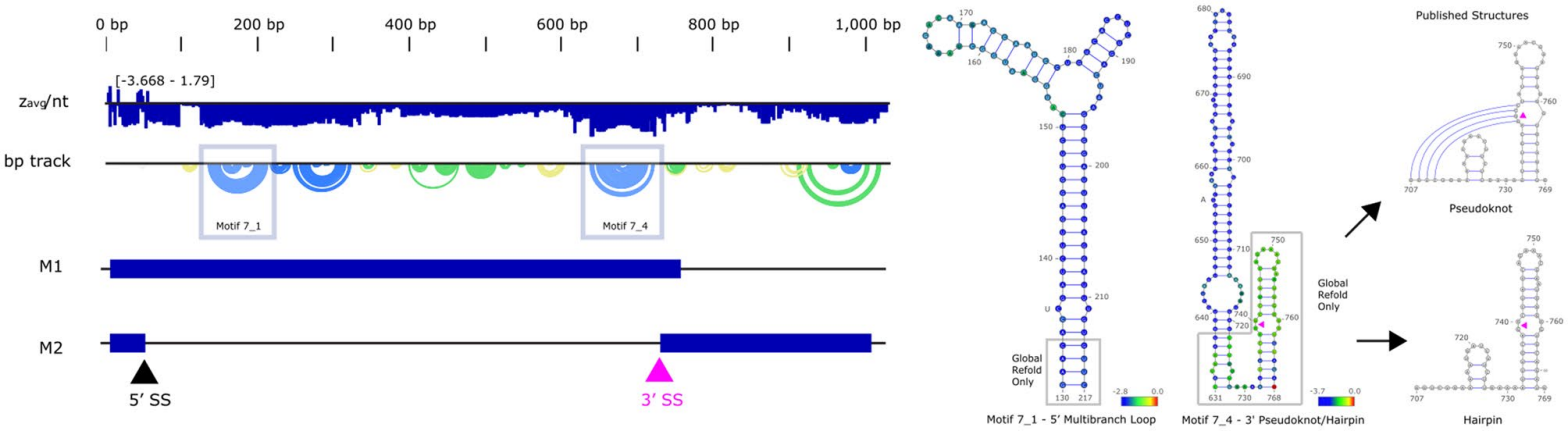
(Left) ScanFold data for IAV 7 ( +), with motif 7_1 and 7_4 designated by a blue box. M1 and M2 versions of this transcript are illustrated for reference. The base pair track (bp track) shows arcs correlating to base pairings, where blue arcs have a <  −2 score, green arcs have a < −1 z-score, yellow arcs have a < 0 z-score, and gray arcs have a z-score > 0. (Right) Motif 7_1 correlates with the published multibranch loop^23^, aligning with our reference sequence after global refold (gray boxes). Motif 7_4 has an extended hairpin that occludes any formation of the 5′ pairing or pseudoknot structure around the 3′ splice site (739–740, purple triangles), but a global refold reveals the published 3′ pairing^25^. Two sequence variations from our reference sequence are annotated at 136 and 655. ScanFold z-scores are overlaid in each nucleotide circle, with blue designating < −2 z-score. Structural images were adapted from VARNA, and the genome illustrations were adapted from NCBI.

Several structural motifs were previously reported for IAV ( +)^14,16–21,23,25^. We were able to recapitulate one of them fully in our current analysis (Fig. 2), a multibranch loop from IAV 7 ( +)^23^. This motif, designated 7_1, has a z_avg_ of -2.31 ± 0.27. While the published structure was from a different sequence than that used to generate our ScanFold data (AF389121.1 vs. NC_002016.1, respectively), the two sequences are 99.4% identical. The ScanFold motif is slightly shorter than the previously published structure (130–217 vs. 134–213), which was predicted using RNAz^33^. The four basal stem base pairs are absent in the ScanFold model, as they fell above the -2 z-score cutoff used to define motifs, and were therefore excluded prior to refold via RNAfold^33^. Notably, if the entire segment is refolded using the low z-score (< −2) structure as a folding constraint, the resulting global model restores these pairs (see “Data availability”). In general, ScanFold motifs are small, as the goal of the program is to identify highly-stable *local* folds.

All but one of the remaining published motifs analyzed contain pseudoknot structures (non-nested base pairs). The folding algorithm used in ScanFold, RNAfold, is unable to predict pseudoknots due to the complexity of non-nested pairing, and instead predicts the nested MFE for a given window. ScanFold predicted motifs near the previously published IAV 7 ( +)^25,34^ pseudoknot/hairpin spanning the 3' splice site of this RNA, but failed to reconstruct the pseudoknot (Fig. 2). This conserved region is vital for the alternative splicing and production of the ion channel protein M2^34^. The pseudoknot and hairpin conformations share two internal pairings (5′, 714–727, and 3′, 732–768) with a non-nested pairing (707–742) forming only in the pseudoknot conformation. Using a -2 threshold to extract motifs, the 5′ and non-nested pairings were overpowered by the upstream motif IAV ( +) 7_4 (637–722), while the 3′ pairing did not meet the threshold. Lowering the threshold to -1, the 3′ pairing can be partially recovered. Further, a global refold at either z-score threshold resulted in a near complete recovery of the 3′ pairing. IAV ( +) 7_4 is able to occlude the 5′ and non-nested pairings due to the structure’s low z_avg_ (-2.78 ± 0.45), whereas the 5′ pairing fell above the default threshold (−1.44 ± 0.63). It should be noted that the initial research that predicted this pseudoknot did not find any low z-score structures in this region; rather, the potential for structure was deduced from analysis of constraints on codon evolution^25^. The pseudoknot was then modeled using DotKnot^35^, which uses pairing probabilities in a heuristic approach for non-nested base pair identification^25^.

Beyond these previously-described motifs, novel structures were also predicted. To assess evolutionary evidence for conserved structure within each motif, we performed covariation analysis. Much of the initial work on structure conservation in influenza virus focused on simple metrics of conservation (e.g., the percent preservation of base pairing across alignments) and highlighted potentially supportive mutations; however, the statistical significance of such variation was not previously assessed. Recently, powerful and user-friendly approaches have emerged for covariation analysis of RNA structure^36–38^, which can identify statistically-significant covariation^39,40^. We performed covariation analysis using the cm-builder pipeline^36^, which chains together the homology discovery suite Infernal^38^ with R-Scape^40^ to provide a robust statistical framework for assessing the potential significance of sequence covariation (structure supporting mutations). Covariance analysis was conducted against a database of 438,519 influenza sequences available from the NCBI Influenza Virus Database (see Materials and Methods).

**Figure 3:**
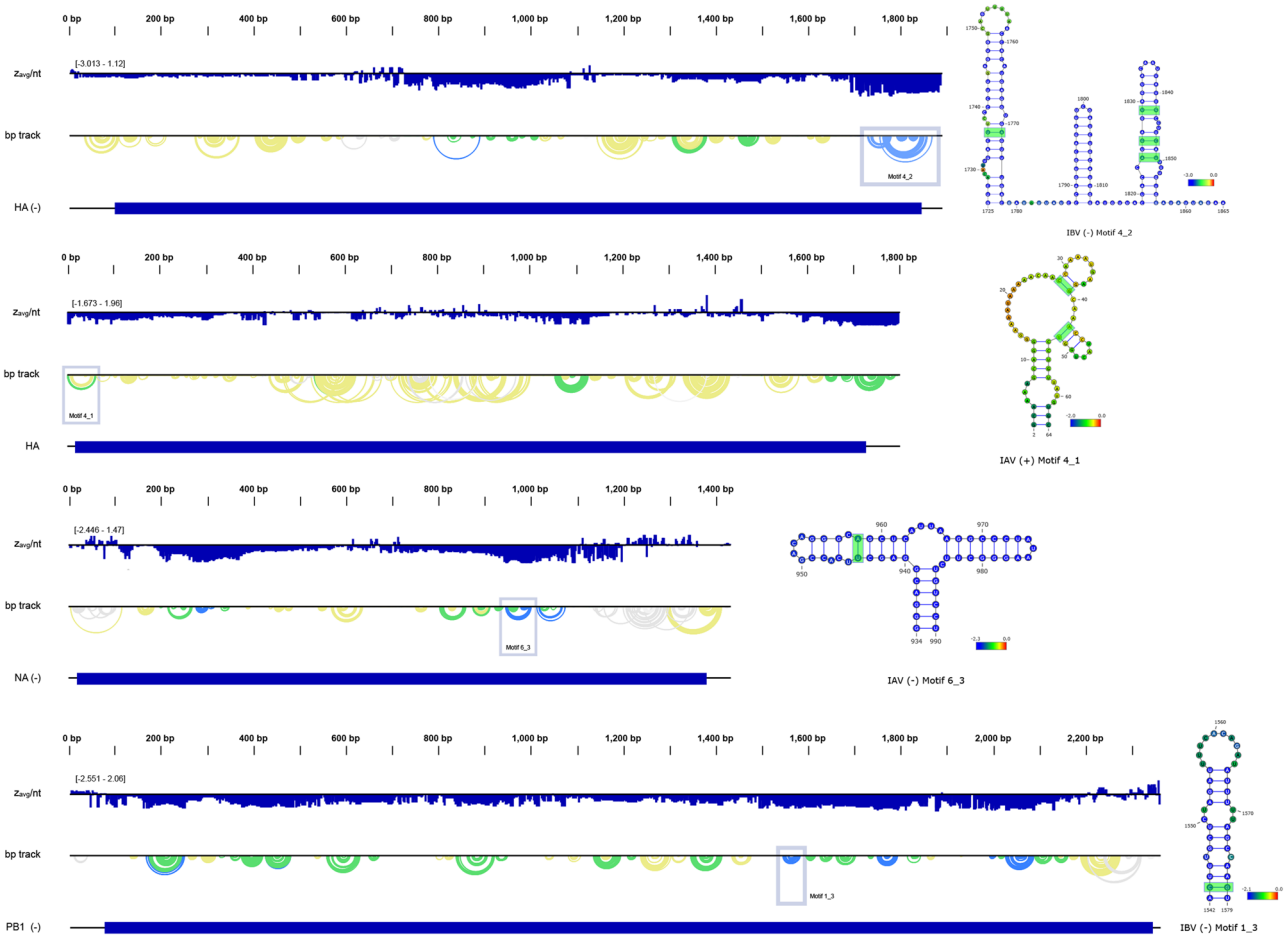
ScanFold analysis for motifs (top to bottom panels, respectively) IAV ( +) 4_1, IAV (−) 6_3, IBV (−) 1_3, and IBV (−) 4_2 (locations designated with blue boxes). The base pair track (bp track) shows arcs correlating to base pairings, where blue arcs have a < −2 score, green arcs have a < −1 z-score, yellow arcs have a < 0 z-score, and gray arcs have a z-score > 0. The R-Scape calculations showed observed base pair covariance (highlighted in green), but the number of observe covarying pairs fell below the expected value (given the sequence alignment). ScanFold per nt z_avg_ are overlaid in each nucleotide circle, with blue designating < −2 z_avg_. Structural images were adapted from VARNA, and the genome illustrations were adapted from NCBI.

**Figure 4:**
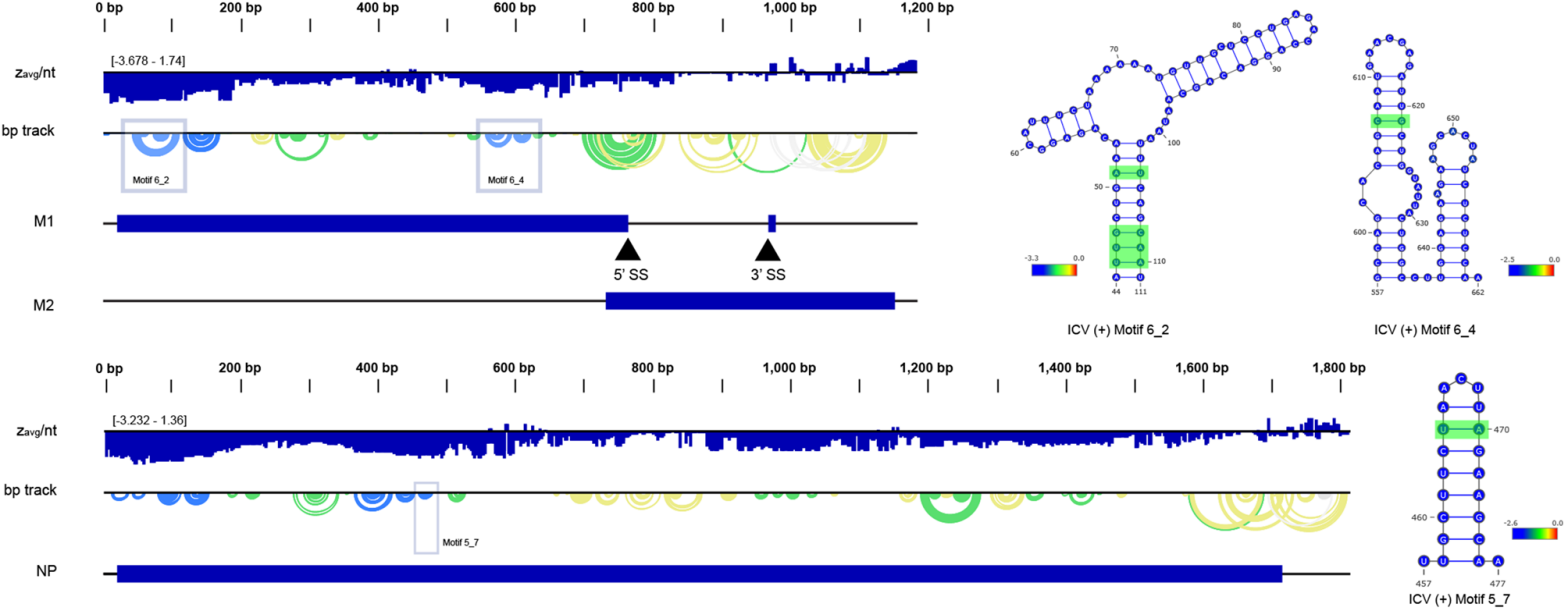
ScanFold analysis for motifs ICV ( +) 6_2 and 6_4 (blue boxes, upper panel) and 5_7 (lower panel). The base pair track (bp track) shows arcs correlating to base pairings, where blue arcs have a < −2 score, green arcs have a < −1 z-score, yellow arcs have a < 0 z-score, and gray arcs have a z-score > 0. The R-Scape results for ICV ( +) 6_2 had 4 bp observed to covary (highlighted in green) with 0.0 ± 0.1 bp expected, while 6_4 had 1 observed bp with 0.0 ± 0.0 bp expected. ICV ( +) 5_7 showed a single observed bp when 0.0 ± 0.2 were expected. ScanFold per nt z_avg_ are overlaid in each nucleotide circle, with blue designating < −2 z_avg_. Structural images were adapted from VARNA, and the genome illustrations were adapted from NCBI.

Only 7 out of 185 low z-score motifs had any covariation identified by R-Scape (examples in Figs. 3 and 4, all motifs available in Supplemental File 2, and covariance data available in Supplemental File 3). Of those, only 3 motifs (ICV ( +) 5_7 vs. all databases, and ICV ( +) 6_2 and 6_4 vs. ICV database) had observed covarying base pairs above the expected number predicted for the input alignment. Covariance calculations for motif IBV (−) 4_2 (1725–1835) showed 4 bp observed to covary with 29.5 ± 1.2 bp expected. The highest number of covarying base pairs were observed in ICV ( +) motif 6_2 (44–111) (Fig. 4); 4 bp were observed with 0.0 ± 0.1 bp expected within the ICV database. ICV ( +) 6_4 (557–662) was predicted to have 1 observed bp against the ICV database, with 0.0 ± 0.0 bp expected (Fig. 4). Only one motif, the 8 bp hairpin ICV ( +) 5_7 (456–477), showed evidence of broad conservation across multiple influenza clades (Fig. 4). ICV ( +) 5_7 showed a single observed covarying base pairing when 0.0 ± 0.2 were expected. These results were based on 24 sequences (13 IAV, 11 ICV), all coding for the segment 5 nucleocapsid protein. All 24 sequences align with our IAV 5 ( +) reference sequence from ~ 1139–1165, with the IAV sequences containing up to an 8-nucleotide insertion not seen in ICV. Interestingly, this insertion aligns within ICV ( +) 5_7’s hairpin loop without disrupting the existing structure. Looking at IAV ( +) 5 in this region (Supplemental File 2), the ordered motif IAV ( +) 5_4 was predicted in this region (1145–1159), but failed to refold as an individual motif due to only consisting of two base pairs. The global refold maintains this motif, however, and can be seen as a very small arc next to IAV ( +) 5_3 (Supplemental File 1).

### Comparison of ScanFold predicted structures to available DMS-MaPseq data

**Figure 5:**
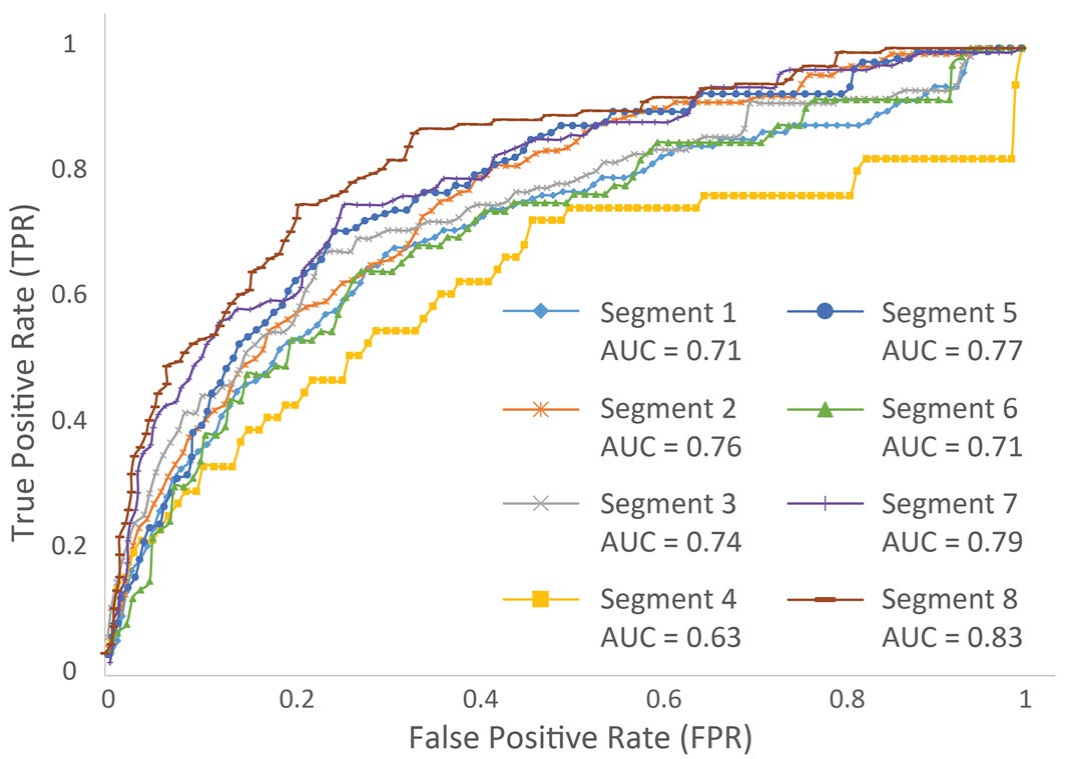
A receiver operating characteristic (ROC) analysis comparing in silico ScanFold −1 z-score predicted structures to DMS-MaPseq data for IAV (H1N1) segments 1–8. The true positive rate (TPR) is shown on the y-axis and the false positive rate (FPR) on the x-axis. Each segment is shown with a unique color and data point marker: segment 1, light blue and a diamond; segment 2, orange and an asterisk; segment 3, grey and an x; segment 4, yellow and a square; segment 5, dark blue and a circle; segment 6, green and a triangle; segment 7, purple and a cross; segment 8, maroon and a dash. The associated area under the curve (AUC) is shown below each segment in the legend.

Using publicly available probing data for IAV (H1N1 strain)^41^, we were able to conduct a receiver operating characteristic (ROC) analysis comparing DMS-MaPseq data to all ScanFold -1 ΔG z-score predicted structures within all 8 positive-sense IAV segments (see “Methods” for greater detail). Briefly, reactivity values are constrained from lowest to highest values at regular (e.g., 1%) intervals and constrained positions are considered to be paired at their corresponding thresholds. Here, constrained DMS-MaPseq datasets were cross referenced to ScanFold predicted structures to yield a true positive rate (TPR) and a false positive rate (FPR) of prediction. The results of this analysis (Fig. 5 and Supplemental File 4) showed that ScanFold predicted structures had a non-random fit and agreed well with the probing data. In an ROC analysis, the area under the curve (AUC) is a measure of how well the data fit and an AUC value of 0.5 would indicate a random fit and a value of 1.0 would indicate a perfect fit. ScanFold predicted structures for all 8 IAV segments had AUCs which ranged from 0.63 for segment 4 and up to 0.83 for segment 8 (Fig. 5).

## Discussion

Influenza RNAs consist of a short (~ 25 nt) untranslated region followed by one large (or multiple overlapping) open reading frame(s). Maintenance of coding potential is a strong evolutionary constraint that can severely limit the available compensatory mutations that also preserve functional RNA structures (e.g., base pairs from wobble sites in codons)^14,21^. In fact, the reciprocal effect of structure on codon use led to the initial discoveries of several elements including the IAV 7 ( +) pseudoknot/hairpin structure^14,25^. Prior research using mutual information, assessing linkages between evolving sites, found signal across several stem-loop structures identified in representative strains of hemagglutinin (segment 4) RNA^17^. This was observed to be most prominent in H5 and H7 subtypes, with varying representation across all 16 subtypes^17^. However, Gultyaev et al. had noted in prior research that it was difficult to maintain significance across all subtypes due to the vast number of influenza variants, and that covariance was most likely subtype-specific^18^. Unfortunately, this hypothesis was not supported by a follow-up analysis using our A/Puerto Rico/8/1934 H1N1 strand against all known IAV H1N1 variants; no covarying base pairs were observed across all segments and strands. It should be noted here that the absence of covariation in RNA structure is not necessarily evidence of a lack of function^37^, and that the work to identify these structures should not be dismissed outright based on this one method.

Given the deep pool of sequences and the ordered structural stability seen across influenza (Fig. 1), the relative scarcity of covariance is initially quite surprising. These findings echo recent debates over the potential covariation in structured long noncoding (lnc)RNAs, where initial analyses using R-Scape found little evidence of covariation in key lncRNAs (such as Xist and HOTAIR), despite numerous studies that supported structure models and functions for them^40,42^. Subsequent work challenged this finding^43^, however the significance of covariation in these RNAs remains a point of contention. Similarly, previous studies posited the existence of conserved structural elements which were (at least for IAV) subjected to subsequent structural probing^20,21,23,24,44–46^ and functional analyses^13,14,17–19,22,25,31^. No motifs with statistical evidence of covariation were found in IAV, and the few hits we did observe were in ICV; indeed, the only motif with wide conservation (across clades) was found in ICV. With this is in mind, it appears that only a few motifs in influenza are evolving under strict structural constraints.

Our previous study of SARS-CoV-2 found similar results in that, despite extensive evidence of ordered stability, only 57 out of 524 motifs showed evidence of covariation^29^. It may be that viral RNA secondary structures can be extensively ordered to fold into stable conformations, but that the evolutionary pressures acting on them are fairly loose. Namely, ordered RNA secondary structural stability may be important for viral function, but *specific* base pairs may not be strongly selected for by evolution. The idea that some viral RNA secondary structures, particularly in influenza, may be under loose structural constraints is supported from recent work on IAV using chemical crosslinking. Extensive long-range intra- and inter-segmental RNA-RNA interactions were identified in IAV using the method 2CIMPL^44^. An interesting finding of this study was that ablating inter-segmental base pairs had less of an impact on viral reassortment than one would predict due to multiple redundant inter-segmental interactions^44^. It may be possible that a similar pattern of redundancy is at play within local influenza RNA structures.

Additionally, our previous SARS-CoV-2 analysis noted that, despite the ScanFold results being purely in silico, they were in agreement with a variety of structure probing data sets (determined via ROC analyses) and that significantly low z-score structures agreed best with probing data^29^. Interestingly, we observe similar levels of agreement of ScanFold predicted structures to available probing data for IAV in this study (via ROC analysis). Furthermore, when previous ScanFold analyses were performed with incorporation of probing data, global trends in the ΔG z-scores were largely unaffected^29^ indicating that the z-score metric can highlight significantly stable regions with or without probing data. Significantly, the z-score metric can highlight interesting trends in the data. For example, in Table 1 there are remarkable biases predicted across different segments/strains. For example, in IAV the two spliced segments (IAV 7 and 8; Table 1) were the only ones to have evidence for global structural ordering (overall z-score < −1 across the sequence) in the ( +)RNA. Notable, in IAV 7 here is a significant strand bias for ordered folding favoring the ( +)RNA that is not the case for IAV 8—suggesting that structure plays more significant roles in the ( +)RNA of IAV 7, potentially for splicing, vs. the genomic (−)RNA. Whereas, in IAV 8 structure could be significant to both the ( +)RNA and (−)RNA; in the latter case, perhaps in genomic packaging. These interpretations are, however, complicated by the lack of global ordering in the spliced segments from IBV and ICV: IBV 8 and ICV 6/7. When focusing on local regions near the splice sites, however, instances of ordered structure were predicted at the 5′ splice sites of IBV 8 (nt 75) and ICV 7 (nt 213) both fall within motifs comprised of z-score < -1 base pairs (Supplemental File 1); however, the 3′ splice sites: IBV 8 nt 731 , ICV 6 nt 753, ICV 6 nt 902, ICV 7 nt 527 nt were not embedded in predicted motifs. One notable limitation of our approach is that ScanFold cannot predict pseudoknots, which were previously proposed for the 3′ splice sites of IBV and ICV^13^. Notably, structural dynamics between pseudoknots and hairpins may also be significant for splicing of influenza; the static weighted-consensus structures of ScanFold would not reflect this either.

Another interesting consideration is the potential roles of ordered structure in constraining influenza sequence evolution. As noted above, the bulk of each genome segment is comprised of coding sequence (sometimes multiple ones), which is a major constraint. Focusing on the 12 low z-score base pairs (< -2) that fell within coding regions, the majority (8/12) had at least one paired nucleotide falling within a wobble position, while 3 base pairs had both nts falling within wobble positions. These observations are in-line with previous work on IAV, which noted localized suppression of synonymous codon usage^47^, which was found to overlap previous predictions of conserved RNA secondary structure^14^, which may be constraining available synonymous substitutions.

## Conclusion

ScanFold provides comprehensive in silico analyses of structure within the three major clades of influenza virus. This work complements previous investigations in its focus on the discovery and advancement of local motifs of interest. While not as structured as SARS-CoV-2, ScanFold analysis shows influenza to have a propensity toward structure on the whole. Further, little covariance within influenza is statistically significant, perhaps owing to the sheer magnitude of similar variants that make covariance a difficult metric for the analysis of influenza^18,42^. The presented report also highlights significantly low z-score regions, which have been shown to correlate well with highly structured sequences^29^. The identification of 185 novel motifs in this work will hopefully lower the barrier to entry for further structure/function analysis of influenza. Further, the motifs provided here, alongside previously described structures, represent high-value targets for additional work to: (i) analyze their functions, (ii) develop 3D models combining computational and biophysical techniques, and (iii) assess their druggability.

## Methods

### ScanFold analysis

Segment nucleotide sequences were downloaded from NCBI for A/Puerto Rico/8/1934 for IAV, B/Lee/1940 for IBV, and C/Ann Arbor/1/50, for ICV (all accession numbers are available in Supplemental File 2). ScanFold^27^ was applied to these sequences, utilizing a 1 nt step, 120 nt window size, 100 randomizations, 37 ˚C on positive and negative strands. These ScanFold parameters have been previously optimized^27,48,49^. All ScanFold Data is available at RNAStructuromeDB^50^.

To focus on local motifs most probable to be structured, the ScanFold 120 nt window, positive and negative strands, <  −2 z-score results were the focus of further evaluation. Motif structures were then extracted, with motifs being considered separate if they had at least two nucleotides between structures. These structures were then refolded via the ViennaRNA package RNAfold^33^, and any structures that completely unfolded were removed from the motif pool. The only exception was IAV 4 ( +), which lacked any < −2 z-score motifs. In this case, the < −1 results were included for covariance analysis. Known motifs (e.g., the IAV 7 ( +) pseudoknot) were also manually added to the motif pool for covariance modeling.

### Covariance

With highly structured motifs now available, the cm-builder script^37^ was used to build a covariance model for each segment and database. This script utilizes Infernal^38^, RNA Framework^36^, and R-Scape^40^ to analyze motifs against sequence databases, resulting in a list of highly structured and highly conserved motifs. The influenza nucleotide databases were downloaded from the NCBI Influenza Virus Database, selecting for each type, filtering for full-length only, and collapsing identical sequences. These sequences were downloaded on 12 January 2021, resulting in 381,893 IAV, 55,958 IBV, and 668 ICV sequences. Each motif was analyzed against an IAV-only, IBV-only, ICV-only database, as well as a database of all available sequences. All resulting covariance models were then compiled (Supplemental File 3), and any observed covariance was assessed for significance (Supplemental File 2). IAV H1N1 segments were downloaded on 9 November 2021 (107,762 sequences), and all IAV H1N1 motifs were tested for covariance; no covariance was observed.

### Receiver operating characteristic analysis of ScanFold predicted structures

ScanFold predicted structures for positive-sense IAV segments which contained −1 ΔG z-score base pairs or lower were cross referenced to available DMS-MaPseq^41^ probing datasets using ROC analysis, which measures how well the predicted model fits the in vivo generated data. In this analysis, reactivity data files (generated by Simon et al.) for each IAV segment had their reactivity sorted from least to most reactive and the lowest values were constrained to be paired at 1% intervals from 0 to 100 percent. Nucleotide positions constrained to be paired are then cross referenced to the predicted ScanFold structure (at every constraint threshold) to determine whether that position is a true positive (TP), false positive (FP), true negative (TN), or false negative (FN) and this is used to determine a true positive rate (TPR) and a false positive rate (FPR) at each threshold. Equations (1) and (2) show the TPR and FPR formulas respectively:

1 $$TPR= \frac{TP}{TP+FN}$$

2 $$FPR= \frac{FP}{FP+TN}$$

Here, a TP occurs when the nucleotide position is paired in the corresponding connectivity table (CT) file and considered paired at the corresponding constraint threshold; a FP occurs when the position is unpaired in the CT file and paired at the reactivity threshold; a TN is unpaired in the CT file and unconstrained at reactivity threshold; and a FN is paired in the CT file and unconstrained at the reactivity threshold. In this way, a completely unconstrained reactivity file, when compared to a CT file, will yield TPRs and FPRs of zero and completely constrained files will yield values of one. If a model fits the corresponding data, the TPR will rise significantly faster than the FPR initially, generating a curve with a larger AUC. If a model is random in regard to the data, the TPR and FPR will rise at an equal rate, generating a roughly 45-degree line. Results of our ROC analysis of IAV are visualized in Fig. 5 and raw data is in Supplemental File 4.

## Supplementary Information

Supplementary Information.

## Data Availability

Influenza ScanFold data is available at the RNAStructuromeDB website: https://structurome.bb.iastate.edu/. Python scripts used in analyses can be found at: https://github.com/moss-lab/.
