# Supplementary material for: In silico analysis of local RNA secondary structure in influenza virus A, B and C finds evidence of widespread ordered stability but little evidence of significant covariation
Source: Sci Rep. 2022 Jan 10;12:310. doi: 10.1038/s41598-021-03767-x (PMC8748542; doi:10.1038/s41598-021-03767-x)
Supplement: Supplementary file 1 — Supplementary Information. [file 41598_2021_3767_MOESM1_ESM.zip › Supplemental Files/README.docx]

**Supplemental Data**

**File 1.** ScanFold bp tracks and nucleotide z-scores for IAV (+/-), IBV (+/-), and ICV (+/-). Blue arcs are base pairs with a z-score of less than -2, green arcs have a z-score from -2 to -1, yellow arcs have a z-score from -1 to 0, and gray arcs are greater than 0. Data are from ScanFold, and images are edited from IGV.

**File 2.** Accession numbers, z_avg_ and total <-2 z-score motifs per segment, as well as motif locations, sequences, and dot bracket notations. Yellow highlights are motifs that unfolded during global refold, and therefore could not be used for covariance analysis. IAV segment 4 (+) <-1 z-scores were included in this analysis. Also includes a summary of all observed covariance, limited to nonzero results and published structure motifs. Green highlights are predicted to be statistically significant, yellow highlights were within the expected standard deviation, and red highlights were not statistically significant.

**File 3.** Folder of cm-builder motifs, including the expected/observed covariance, power outputs, and PDF structures. Stockholm files for all nonzero covariance results are also included. Published structure data are also included, despite all covariance results being zero. “CM” is covariance model, followed by a symbol combination designating the origin of the motif the database used (“A” for IAV, “B” for IBV, “C” for ICV, “All” for IAV, IBV and ICV, “Pub” for published motifs, “rev” for negative strand database).

**File 4.** Raw data from the receiver-operating characteristic (ROC) analysis and the associated file names used to generate TPR and FPR data. Here, ScanFold -1 (and lower) z-score structures were compared to DMS-MaPseq data for the IAV H1N1 strain. Descriptions of data columns are present in the file.
